# The Prognostic Value of Baseline Neutrophil-to-Lymphocyte Ratio for Mortality in Idiopathic Pulmonary Fibrosis: A Systematic Review and Meta-Analysis

**DOI:** 10.7759/cureus.116706

**Published:** 2026-09-22

**Authors:** Suprava Shakya, Xingxiang Xu

**Affiliations:** 1 Department of Respiratory Medicine, Northern Jiangsu People's Hospital Affiliated to Yangzhou University, Yangzhou, CHN; 2 Department of Respiratory Medicine, Soochow University BenQ Medical Center, Suzhou, CHN

**Keywords:** idiopathic pulmonary fibrosis, ipf, meta-analysis, mortality, neutrophil-to-lymphocyte ratio, nlr, prognosis, systematic review

## Abstract

Idiopathic pulmonary fibrosis (IPF) is a chronic fibrosing pulmonary disease with an unfavorable prognosis. It usually occurs in the older age group and shows an extremely variable clinical course. Neutrophil-to-lymphocyte ratio (NLR) is an affordable, widely accessible blood-based biomarker that has great potential for evaluating disease outcomes. Therefore, this systematic review and meta-analysis aim to synthesize the existing literature and analyze the prognostic value of baseline NLR for mortality in IPF patients. Literature was retrieved from PubMed, Web of Science, Embase, and ScienceDirect from the earliest available records to April 2026. Studies investigating the prognostic role of baseline NLR in IPF patients and reporting mortality outcomes with HRs were included. Random-effects (RE) and fixed-effects (FE) models were used to pool HRs. The Newcastle-Ottawa Scale (NOS) was used to assess the quality of included studies. This meta-analysis included four retrospective cohort studies comprising 1,524 IPF patients. All studies demonstrated good quality (NOS 8/9). Pooled analysis indicated that increased baseline NLR was related to increased mortality risk (pooled HR = 1.54, 95% CI 0.90-2.64, p = 0.083), although statistical significance was not reached under the RE model. The FE model was used as a sensitivity check, yielding a similar direction of association (HR 1.43, 95% CI 1.21-1.68), although the RE model was prioritized due to observed heterogeneity. Furthermore, a supplementary exploratory analysis demonstrated a pooled HR of 1.67 (95% CI 1.12-2.49) under the RE model, with the association remaining in the same direction as the primary analysis. However, these analyses were exploratory, and findings should be interpreted with caution as they combine adjusted and unadjusted estimates. The leave-one-out sensitivity analysis further supported the stability of our pooled results. This systematic review and meta-analysis suggest that an elevated baseline NLR is associated with a nonsignificant trend toward increased mortality risk in IPF patients, as the primary RE model did not reach statistical significance. Prospective multicenter studies with standardized NLR cutoff thresholds are needed to verify its clinical application.

## Introduction and background

Idiopathic pulmonary fibrosis (IPF) is a chronic, progressive fibrosing interstitial lung disease of unknown etiology that predominantly affects older adults and is characterized by a highly variable clinical course. It is marked by histological and radiological findings consistent with usual interstitial pneumonia (UIP), leading to progressive respiratory decline and an unfavorable prognosis [1]. The majority of patients experience increased cough, dyspnea, and a notable decline in their quality of life [2]. Although antifibrotic therapy is available to slow disease progression [3,4], prognosis remains poor, with a median survival duration of roughly two to three years in the absence of treatment [2,5]. The disease’s clinical course is highly unpredictable, as there may be very slow disease progression in some cases, while in others there may be rapid deterioration causing respiratory failure and death [6].

By virtue of the disease’s uncertainty, risk stratification models such as the Gender-Age-Physiology (GAP) index are utilized for predicting patients’ prognosis; it incorporates gender, age, and physiological measurements such as diffusing capacity of the lungs for carbon monoxide (DLCO) and forced vital capacity (FVC), which serve as references for illness severity [7]. However, they have their own limitations, as they may overestimate risk in some populations and do not incorporate exercise parameters that may influence outcomes [8]. Also, the GAP index can be challenging to implement in frail patients, as it relies on FVC and DLCO measurements obtained via pulmonary function tests [7]. These measurements are often uninterpretable in frail patients [9], and their prognostic value also seems to be limited in elderly IPF patients [10]. Therefore, more accessible blood-derived biomarkers are needed to improve current risk stratification.

In this context, as studies suggest inflammation and immune dysregulation contribute significantly to the etiology of IPF [11], the neutrophil-to-lymphocyte ratio (NLR), calculated as the ratio of neutrophil to lymphocyte count [12], has shown potential as a promising, economical, accessible prognostic marker for evaluating systemic inflammation and identifying vulnerable patients with IPF [13]. Reportedly, many diseases, like chronic obstructive pulmonary disease, malignancies, rheumatoid arthritis, and cardiovascular diseases, have been correlated with NLR [14-17]. This observation has further increased interest in exploring the utility of NLR as an outcome-related biomarker in IPF.

Following this, multiple studies have explored the role of baseline NLR in predicting outcomes among IPF patients, demonstrating its association with mortality, disease progression, and transplant-free survival [18-22]. Therefore, this systematic review and meta-analysis aims to synthesize current evidence and clarify the prognostic value of baseline NLR in patients with IPF.

## Review

Methods

We followed the principles of the PRISMA 2020 guidelines [23]. However, we did not register this study in PROSPERO because it was conceptualized as an exploratory, hypothesis-generating review, not a confirmatory one with a predefined protocol. Still, we strongly encourage prospective registration for systematic reviews and meta-analyses to improve transparency and reduce selective reporting.

Literature Search

We systematically searched PubMed, Web of Science, Embase, and ScienceDirect from the earliest available documents until April 2026. We did not employ any filters or restrictions during the search. We used database-specific controlled vocabulary and relevant keywords combined with Boolean operators in the literature search. We retrieved other relevant studies by manually screening the reference lists of associated papers. However, trial registries, conference proceedings, and other gray literature were not systematically searched.

Eligibility Criteria

We included studies that fulfilled the listed criteria: (1) studies enrolling IPF patients over 18 years with a diagnosis confirmed by established international guidelines, including American Thoracic Society/European Respiratory Society/Japanese Respiratory Society/Latin American Thoracic Association consensus criteria or diagnosis based on high-resolution CT showing UIP pattern or multidisciplinary discussion where available; (2) studies evaluating baseline NLR (measured at the time of IPF diagnosis or study enrollment) as a prognostic marker; (3) studies reporting HRs with 95% CI or sufficient data to calculate an HR; (4) randomized controlled or observational cohort studies (prospective or retrospective); (5) human studies; and (6) full-text articles available.

We excluded studies that met any of the listed criteria: (1) studies not specific to IPF (e.g., mixed interstitial lung disease without separate IPF data); (2) studies not evaluating NLR; (3) studies not reporting mortality outcomes; (4) case reports, conference abstracts, review articles, meta-analyses, animal/laboratory research, letters, editorials; (5) duplicate or overlapping datasets; and (6) studies with insufficient data to extract effect estimates.

Study Selection 

Following a thorough database search, we identified 185 records. We removed duplicate records using Zotero, which left us with 118 articles. We then screened titles and abstracts and excluded 95 unrelated articles. We reviewed the remaining 23 full-text articles for eligibility. After this, five studies met our criteria, and we included them in the final meta-analysis (Figure 1).

**Figure 1:**
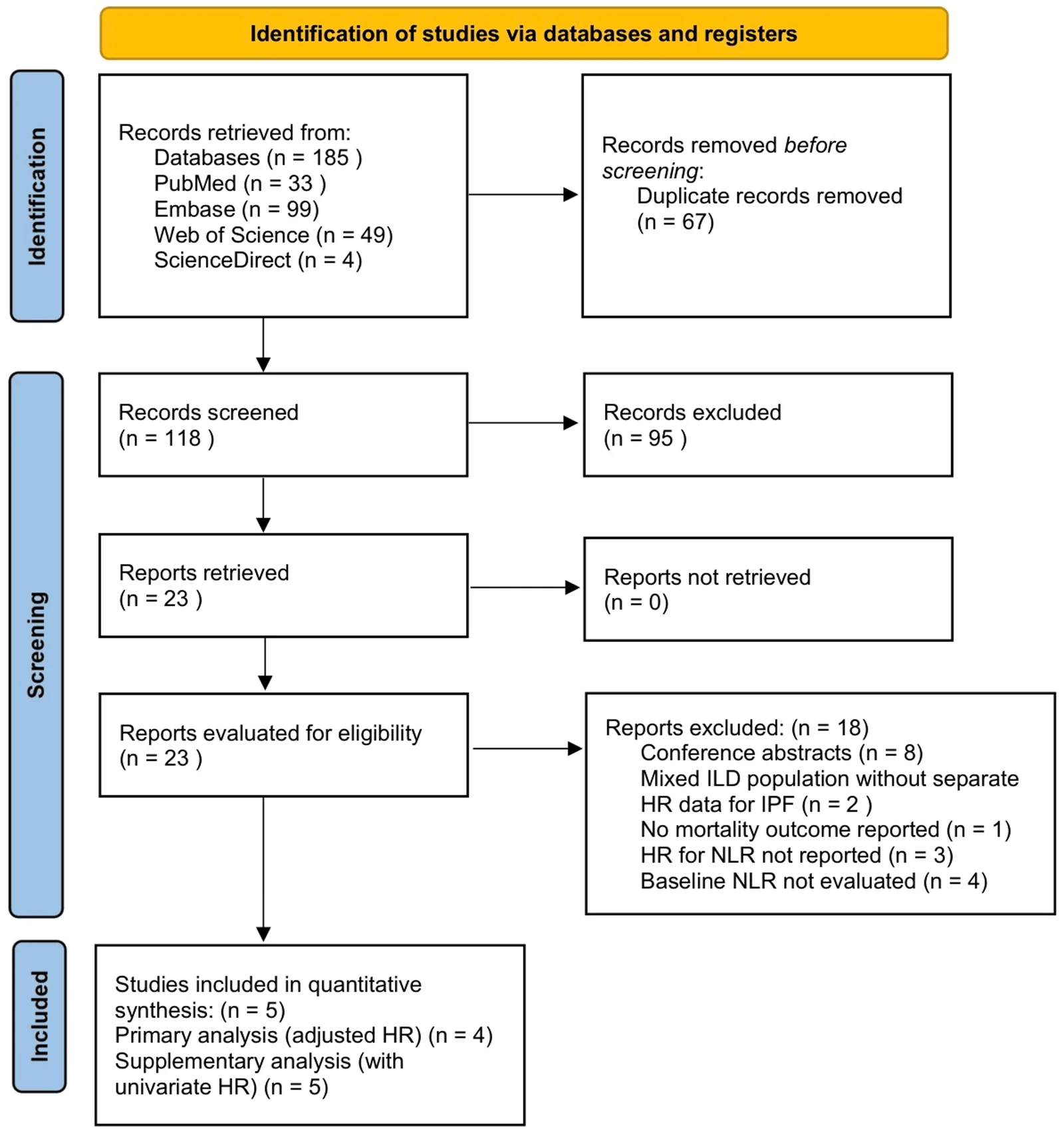
PRISMA 2020 flow diagram of the literature search and study selection process. Studies included in the primary analysis (adjusted HRs): Mikolasch et al. [18], Polat et al. [19], Achaiah et al. [20], and Chen et al. [21]. The supplementary exploratory analysis additionally included Barratt et al. [22] (unadjusted HR) to assess directional consistency; this estimate was not pooled in the primary analysis to avoid combining adjusted and unadjusted estimates. ILD, interstitial lung disease; IPF, idiopathic pulmonary fibrosis; NLR, neutrophil-to-lymphocyte ratio

The first author (SS) performed the title and abstract screening and full-text review. We resolved any uncertainties through discussion with the senior author (XX). We included four studies with adjusted HRs in the primary analysis [18-21]. We also included one additional study reporting an unadjusted HR [22] in a supplementary exploratory analysis, the rationale for which is detailed in the Statistical analysis section.

Data Extraction

The first author (SS) performed the data extraction, and the senior author (XX) checked it for accuracy. We obtained relevant data from each included study, including the authors, country, year of publication, research design, number of participants, patient characteristics, NLR cutoff value, HRs with 95% CIs, adjusted covariates, follow-up period, and outcome definition (Table 1, Table 2). We resolved any disagreements through consensus and discussion.

**Table 1:** Characteristics of the included studies in the primary analysis. apoB, apolipoprotein B; CEA, carcinoembryonic antigen; CYFRA21-1, cytokeratin 19 fragment; DLCO, diffusing capacity of the lungs for carbon monoxide; FVC, forced vital capacity; LDH, lactate dehydrogenase; NLR, neutrophil-to-lymphocyte ratio; NSE, neuron-specific enolase; PaO₂/FiO₂, ratio of arterial oxygen partial pressure to fractional inspired oxygen; RDW, red cell distribution width; TC, total cholesterol; TLCO, transfer factor of the lung for carbon monoxide

Study	Year	Country	Study design	Sample size	Mean age	Male (%)	NLR cutoff	Adjusted HR (95% CI)	Follow-up (months)	Adjusted covariates	Outcome
Mikolasch et al. [18]	2022	UK	Multicenter retrospective	999	72.7	76.9	≥2.9	1.36 (1.12-1.66)	44.4	Age, sex, FVC%, TLCO%, steroids	Transplant-free survival
Polat et al. [19]	2024	Turkey	Single-center retrospective	119	67.9	80.7	≥2.67	2.68 (1.48-4.85)	56.8	RDW, BMI, DLCO	All-cause mortality
Achaiah et al. [20]	2022	UK	Single-center retrospective	128	75.22	78.1	>2.77	1.81 (1.01-3.23)	31	Gender, age, baseline FVC%	All-cause mortality
Chen et al. [21]	2022	China	Single-center retrospective	278	67.97	79.14	≥3.65	1.15 (0.75-1.75)	5.84	Gender, age, smoking, PaO₂/FiO₂, CEA, TC, WBC, LDH, apoB, CYFRA21-1, NSE	Overall survival

**Table 2:** Characteristics of the additional study included in supplementary exploratory analysis. NLR, neutrophil-to-lymphocyte ratio

Study	Year	Country	Study design	Sample size	Mean age	Male (%)	NLR cutoff	Univariate HR (95% CI)	Outcome
Barratt et al. [22]	2021	UK	Multicenter retrospective	520	75	74	>2.19	2.10 (1.54-2.81)	All-cause mortality

Quality Assessment

As all selected studies were retrospective observational studies, we evaluated their quality using the Newcastle-Ottawa Scale (NOS) for cohort studies (Table 3). It evaluates three components: selection (maximum 4 stars), comparability (maximum 2 stars), and outcome (maximum 3 stars). Studies scoring between 6 and 9 stars were considered good quality, whereas a score of 5 or lower was considered lower quality. The NOS was selected because all included studies were retrospective cohort studies, and the NOS is a widely used and validated tool for assessing the quality of non-randomized studies in meta-analyses. The first author (SS) performed the quality assessment, and any disagreements were resolved through discussion with the senior author (XX).

**Table 3:** NOS quality evaluation of included studies. NOS, Newcastle-Ottawa Scale

Study	Year	Selection (max 4)	Comparability (max 2)	Outcome (max 3)	NOS score	Quality
Mikolasch et al. [18]	2022	***	**	***	8	Good
Polat et al. [19]	2024	***	**	***	8	Good
Achaiah et al. [20]	2022	***	**	***	8	Good
Chen et al. [21]	2022	***	**	***	8	Good
Barratt et al. [22]	2021	***	**	***	8	Good

Statistical Analysis

We used R software (version 4.6.2) with the “meta” package for all statistical analyses. We extracted adjusted HRs and their 95% CIs. When studies reported both univariate and multivariate HRs, we used multivariate estimates for primary pooling. However, one study reported only univariate HR [22]. We excluded this from the primary analysis but retained it in a supplementary exploratory analysis to maximize the available evidence for this specific population. The supplementary analysis combined adjusted and unadjusted estimates, which can introduce bias through residual confounding and limit comparability. However, we performed it only to assess directional consistency, not to provide confirmatory evidence of an association between NLR and mortality. It was explicitly exploratory, not confirmatory, and we did not include it in our primary inferential analysis.

We calculated pooled HRs with their 95% CIs. We evaluated statistical heterogeneity among studies using I² statistics and Cochran’s Q test, where I² = 0-25% denoted low heterogeneity, I² = 25-50% denoted mild heterogeneity, I² = 50-75% denoted moderate heterogeneity, and I² > 75% denoted substantial heterogeneity [24]. For the random-effects (RE) model, we estimated the between-study variance (τ²) using the restricted maximum likelihood (REML) method. We used an RE model for the primary analysis because the studies included were quite different from one another (e.g., varying NLR cutoffs, patient populations, and follow-up durations). However, we also ran a fixed-effects (FE) model just as a sensitivity check to see if the results were consistent.

A leave-one-out sensitivity analysis was done to examine factors contributing to heterogeneity and to evaluate the stability of the results. Publication bias was evaluated using Egger’s test and a funnel plot. However, with only four studies included, these results were interpreted with caution and were done only for completeness, as publication bias assessment typically requires a larger number of studies (usually n > 10) for robust interpretation [25-27]. All statistical tests were two-sided, with p < 0.05 considered statistically significant.

Results

Four studies were included in the primary quantitative meta-analysis. Among them, three studies reported a significant association when NLR was analyzed as a dichotomized variable [18-20], whereas one study by Chen et al. reported a nonsignificant association [21], showing an HR of 1.146 (95% CI: 0.75-1.75) using a cutoff of ≥3.65 [21]. However, when the same author analyzed NLR as a continuous variable per 1 SD increase, it was statistically significant, suggesting that the choice of cutoff may have influenced their categorical findings [21].

We pooled four studies with adjusted HRs using a RE REML model with Knapp-Hartung adjustment. The pooled HR was 1.54 (95% CI 0.90-2.64; p = 0.083). Heterogeneity was 55.1% by I², with a Q-test p-value of 0.101 (Figure 2). We also ran an FE model to check for robustness, which produced a pooled HR of 1.43 (95% CI 1.21-1.68), indicating that the direction of association was consistent with that of the primary RE model. However, as the studies were somewhat heterogeneous, we used the RE model as our primary result.

**Figure 2:**
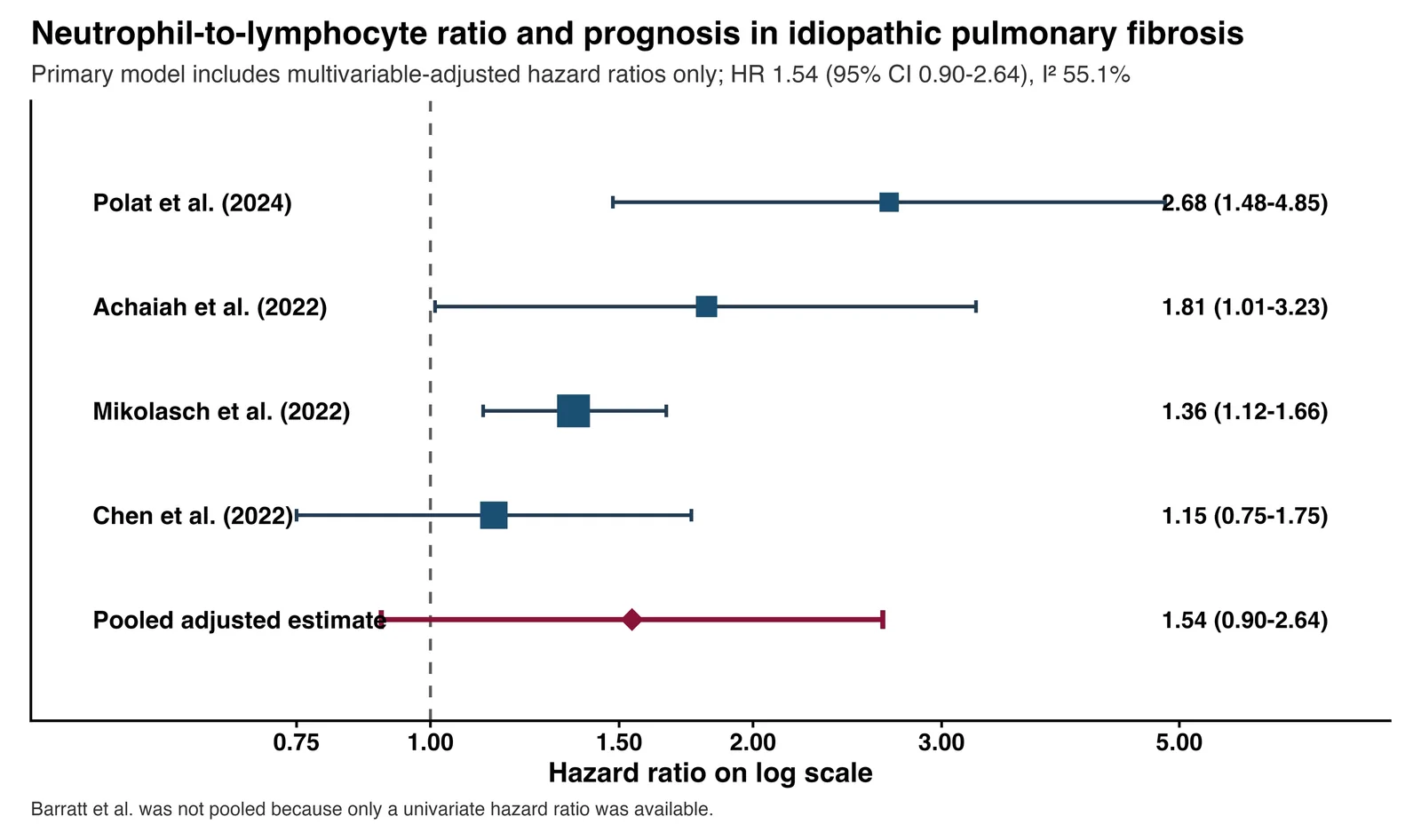
Forest plot of the primary adjusted HR meta-analysis. Four studies (Mikolasch et al. [18], Polat et al. [19], Achaiah et al. [20], and Chen et al. [21]) with adjusted HRs were pooled. Barratt et al. [22] was not pooled because only a univariate HR was available.

A supplementary exploratory analysis including the unadjusted HR from Barratt et al. [22] yielded a pooled HR of 1.67 (95% CI 1.12-2.49) under the RE model and 1.56 (95% CI 1.35-1.80) under the FE model (Table 4, Appendix A).

**Table 4:** Results of the primary and supplementary meta-analyses using RE and FE models. The primary analysis included studies with adjusted HRs [18-21], whereas the supplementary analysis included one additional study, Barratt et al. [22], with unadjusted HRs for exploratory purposes. FE, fixed-effects; RE, random-effects; REML, restricted maximum likelihood

Analysis set	Model	Studies	Pooled HR (95% CI)	p-value	I² (%)	τ²	Q test p
Primary adjusted-only	RE REML with Knapp-Hartung	4	1.54 (0.90-2.64)	0.083	55.1	0.053	0.101
Primary adjusted-only	Fixed-effect inverse-variance	4	1.43 (1.21-1.68)	<0.001	51.9	0	0.101
Supplementary five-study exploratory	RE REML with Knapp-Hartung	5	1.67 (1.12-2.49)	0.024	64.3	0.061	0.025
Supplementary five-study exploratory	Fixed-effect inverse-variance	5	1.56 (1.35-1.80)	<0.001	64	0	0.025

A leave-one-out analysis was used because the evidence base was too small for a meaningful subgroup analysis or meta-regression. The pooled point estimate remained above 1.0 in all omission scenarios, ranging from 1.36 to 1.74. CIs widened in some scenarios, which was expected when only four adjusted estimates were available (Table 5, Figure 3). A supplementary analysis, including Barratt et al. [22], also showed the pooled HR above 1.0 across all omission scenarios. However, given that this analysis combined adjusted and unadjusted HRs, the appropriate interpretation was therefore directional consistency, rather than a claim of definitive certainty (Appendix B, Appendix C).

**Table 5:** Leave-one-out sensitivity analysis for the primary adjusted meta-analysis.

Omitted study	Pooled HR (95% CI)	p-value	I² (%)
Mikolasch et al. [18]	1.71 (0.59-5.00)	0.164	62.4
Polat et al. [19]	1.36 (0.97-1.89)	0.058	0
Achaiah et al. [20]	1.52 (0.55-4.17)	0.216	71.6
Chen et al. [21]	1.74 (0.75-4.04)	0.107	59.2

**Figure 3:**
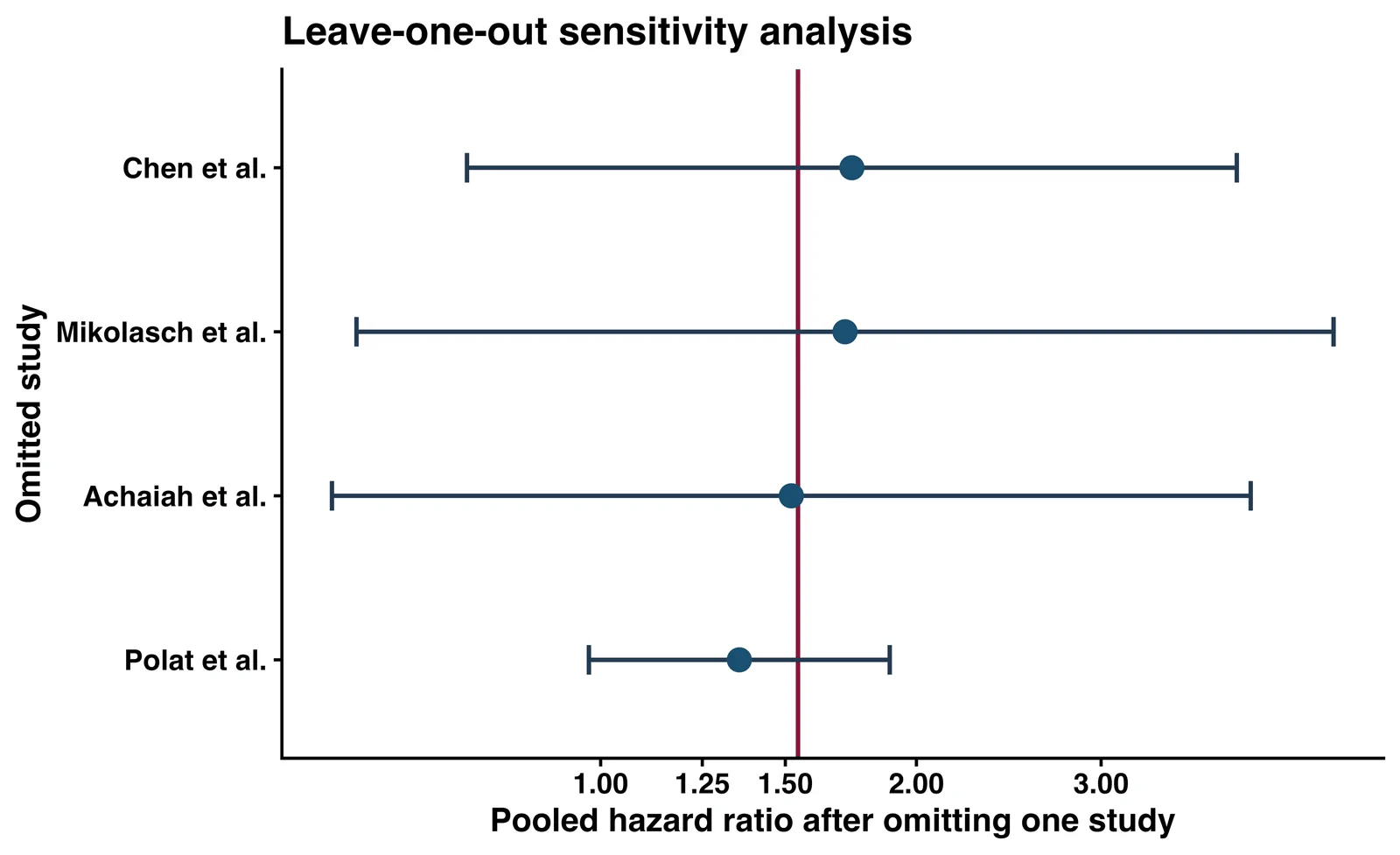
Leave-one-out sensitivity analysis for the adjusted HR. The analysis included Mikolasch et al. [18], Polat et al. [19], Achaiah et al. [20], and Chen et al. [21].

We used a funnel plot and Egger’s regression analysis to look for potential publication bias. For the primary analysis of four studies, Egger's test gave a p-value of 0.353. For the supplementary analysis of five studies, it was 0.461. Because we only had four adjusted studies, this test has very low power and should not be used to rule out publication bias. However, we did this assessment only for completeness, as formal small-study-effect inference remains unreliable when the number of studies included is below 10 (Table 6, Figure 4, Appendix D).

**Table 6:** Publication bias assessment using Egger’s regression test. The primary analysis included adjusted HRs from [18-21]. The supplementary exploratory analysis additionally included the unadjusted HR from Barratt et al. [22].

Analysis set	Test	Studies	Statistic	p-value	Interpretation
Primary adjusted-only	Egger regression test	4	1.2	0.353	Performed for completeness; interpret cautiously because fewer than 10 studies were available.
Supplementary five-study exploratory	Egger regression test	5	0.84	0.461	Exploratory only because adjusted and univariate HRs were combined and fewer than 10 studies were available.

**Figure 4:**
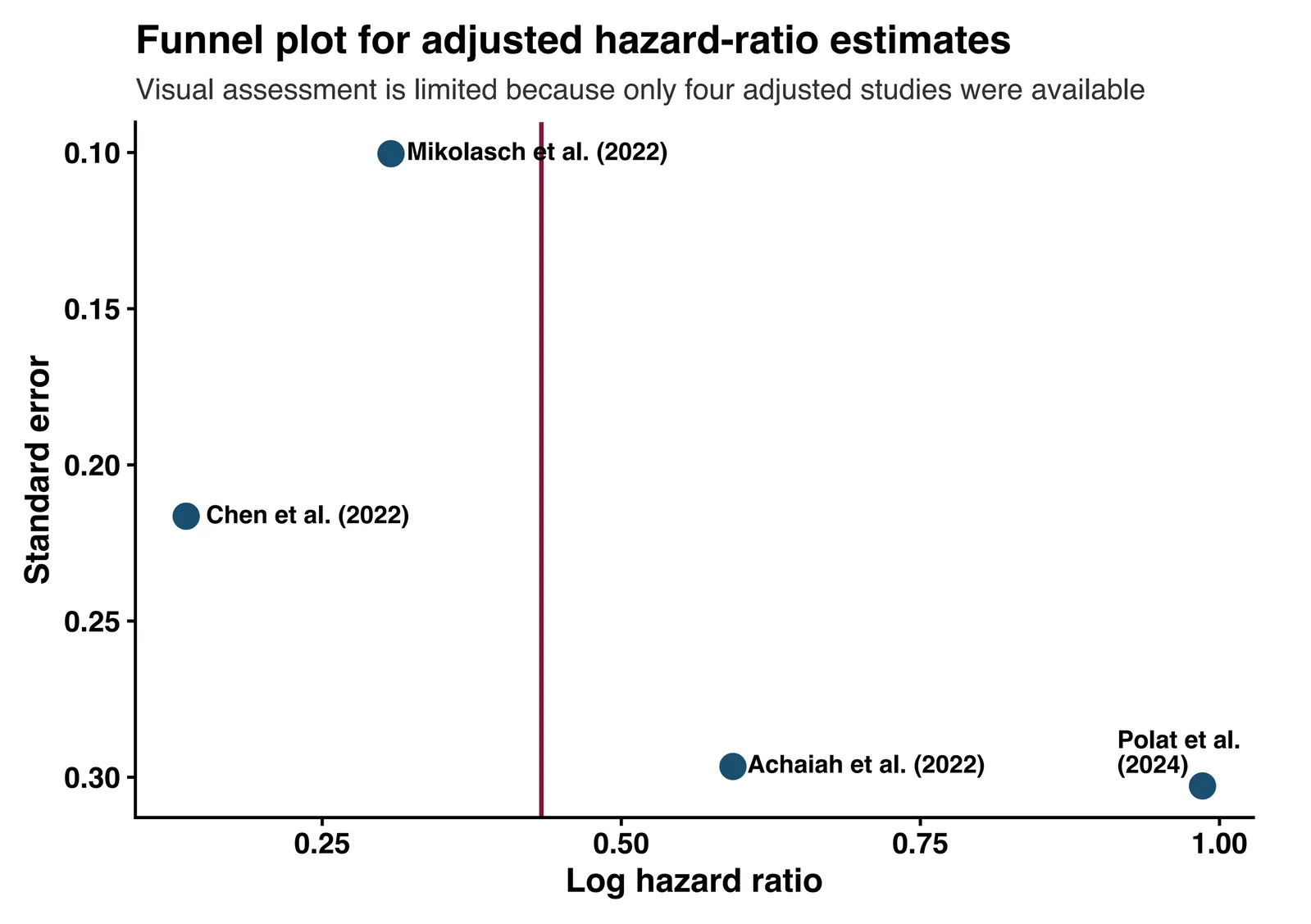
Funnel plot for adjusted HR estimates. Estimates were derived from Mikolasch et al. [18], Polat et al. [19], Achaiah et al. [20], and Chen et al. [21]. Interpretation is limited due to the small number of eligible studies.

Discussion

Biological Rationale

IPF is a pulmonary disease marked by repetitive alveolar epithelial injury and dysregulated repair processes, which activate fibroblasts and myofibroblasts. These activities lead to deposition of excessive extracellular matrix, thickening of the alveolar wall, and impaired pulmonary gas exchange [28-30]. Beyond these pathological mechanisms, emerging evidence suggests that innate and adaptive immunity dysregulation has significantly influenced disease progression and mortality in IPF. Several immune cell populations, such as neutrophils and lymphocytes, have been identified as key contributors to systemic inflammation, where a high NLR often signals an immune system imbalance that is associated with poorer clinical outcomes [13].

Neutrophils are among the first inflammatory cell types to be drawn to injured lung tissue and, upon activation, release a variety of cytokines, chemokines, neutrophil extracellular traps, and neutrophil elastase, which promote fibroblast activation, extracellular matrix remodeling, and fibrotic tissue deposition [11,31]. Although prominent neutrophilic infiltration is not a characteristic feature of classic interstitial lung disease, accumulating evidence suggests neutrophils contribute to IPF pathogenesis through profibrotic mediators [11]. Concurrently, a dysregulated lymphocyte response leads to systemic immune impairment that drives disease progression. Although the function of lymphocytes in IPF is not yet fully understood, Th2 and Th17 cells facilitate fibrosis through profibrotic cytokines, like TGF-β1, IL-4, and IL-13, whereas diminished regulatory T-cell function and reduced interferon-γ levels may compromise immune homeostasis, thereby promoting disease progression [32]. Thus, a high NLR might work as a biomarker of both systemic inflammatory activity and underlying dysregulation of the adaptive immune response, both of which are related to adverse clinical outcomes in IPF.

Interpretation of Findings

The primary analysis included 1,524 patients and produced a pooled HR of 1.54, suggesting a trend toward higher mortality risk with elevated NLR. But it did not reach statistical significance under the RE model. We also ran an FE model and a supplementary exploratory analysis including the unadjusted HR from Barratt et al. [22], both of these yielded estimates in the same direction. However, we ran the FE model only as a sensitivity check, and the supplementary analysis combined adjusted and unadjusted estimates, which could introduce residual confounding. So, we based our main interpretation on the primary adjusted analysis.

Comparison With Previous Studies

The direction of the pooled estimate was generally consistent with the findings of individual studies included in the quantitative synthesis. Mikolasch et al. found that an NLR greater than 2.9 in patients with IPF was linked to significantly poorer survival and higher GAP scores [18]. This suggests that an elevated NLR may reflect both advanced disease severity and adverse clinical outcomes [18]. Achaiah et al. found that a high baseline NLR independently relates to both faster FVC decline and increased all-cause mortality among IPF patients [20]. Consistent with these findings, Polat et al. [19] and Barratt et al. [22] also found a similar relationship between high NLR and poorer survival outcomes. Among the included studies, Chen et al. reported a nonsignificant association when NLR was analyzed using a categorical cutoff [21]. However, their continuous per 1 SD analysis was statistically significant [21]. This suggests the differences may be due to methodological differences, a shorter follow-up period (5.84 months), and a higher NLR cutoff, which could have resulted in nonsignificant associations in their categorical analysis [21].

Beyond predicting mortality, multiple studies have linked the NLR to disease severity and progression in IPF. Zinellu et al. observed that NLR was markedly elevated among IPF patients relative to normal cohorts, leading to reduced FVC and DLCO, indicating a potential relationship between systemic inflammation and diminished lung function [33]. In a study by Nathan et al., longitudinal elevations in NLR over a 12-month duration are independent, reliable predictors of adverse clinical prognosis [34]. Also, studies show that an increased NLR at the start of antifibrotic treatment, specifically pirfenidone, is associated with a less favorable prognosis among IPF patients [35]. Overall, these findings support our results and highlight the potential of NLR as a predictor of clinical outcomes in IPF.

We also identified Ay et al. during our literature search [36], who found significant cross-sectional correlations between NLR and FVC, 6MWD, and GAP index among 155 IPF patients. However, they did not find a significant association between NLR and mortality (p = 0.116). Because it did not report an HR or sufficient data to calculate one, we could not include this study in our quantitative synthesis. Thus, these inconsistencies between cross-sectional and mortality findings indicate that we need more research to clarify NLR’s prognostic role.

Methodological Heterogeneity

The studies included in this review showed considerable heterogeneity. The NLR cutoff values ranged from 2.19 to 3.65 (Table 1, Table 2), which reflects variability in study populations, laboratory assays, and statistical approaches used to determine optimal cutoffs. Furthermore, outcome definitions also varied among included studies: Mikolasch et al. used transplant-free survival [18], while the other included studies used all-cause mortality or overall survival [19-22]. These methodological differences may have contributed to the observed heterogeneity. So, while the NLR shows promise as a prognostic biomarker, the lack of standardized cutoff values and methodological consistency across studies limits its applicability in routine clinical practice and emphasizes the need for future prospective, multicenter studies to establish and confirm a standard cutoff.

Clinical Implications

The NLR is a cost-effective and readily available biomarker obtained from WBC differential counts [12,13,18]. Compared with more complex prognostic models such as the GAP index, NLR provides a simpler means of monitoring patients over time, drawing on routine blood test data to stratify individuals according to their level of risk [18]. Originally proposed by Zahorec [37] as a marker of severity, higher NLR values denote a pro-inflammatory state and have been correlated with adverse clinical outcomes in chronic inflammatory conditions such as IPF [33,35]. Furthermore, a higher baseline NLR may prompt more intensive clinical surveillance, earlier consideration of lung transplantation referral, and more individualized management strategies [13,18,35]. The direction of our findings aligns with Dong et al. [24], who found higher NLR values in interstitial lung disease patients with poor outcomes. However, unlike Dong et al. [24], who evaluated a heterogeneous ILD population, our analysis focuses exclusively on IPF patients, thereby providing more precise prognostic thresholds for this specific fibrotic subtype.

To our knowledge, no previous systematic review and meta-analysis has specifically explored the prognostic utility of baseline NLR for mortality in IPF. All the included studies were of good quality as assessed by NOS. The leave-one-out sensitivity analysis also confirmed the stability of our pooled estimates, as the HR remained above 1.0 in all omission scenarios. One included study, Chen et al. [21], reported a nonsignificant categorical analysis. However, when we excluded this study, the pooled estimate did not change much, suggesting that our overall findings were unaffected by its removal. Thus, by combining the current evidence, our meta-analysis provides a broader picture of NLR’s prognostic value in IPF patients than any single study could alone.

Limitations

However, this systematic review and meta-analysis has several limitations. First, all eligible studies included in this meta-analysis were retrospective in design, and quality assessment was performed using NOS, which may not comprehensively capture domain-specific biases, such as residual confounding, selection bias, or outcome measurement bias. Second, only four studies were available for primary quantitative synthesis, which may limit the statistical power, preclude meaningful subgroup analyses or meta-regression to explore heterogeneity, and contribute to variability in NLR cutoffs, patient population, and outcome definitions. Consequently, the assessment of publication bias using funnel plot asymmetry tests and Egger’s regression has negligible power with fewer than 10 studies and should not be interpreted as an absence of publication bias; these results are presented for completeness only [26,27]. Third, the supplementary exploratory analysis combined adjusted and unadjusted HRs, which can introduce bias through residual confounding and limit the comparability of pooled estimates. Therefore, this analysis was explicitly exploratory and hypothesis-generating rather than confirmatory.

Fourth, the first author (SS) carried out the screening and data extraction, with all extracted data and final study selections subsequently verified and approved by the senior author (XX). While the senior author supervised this process, we did not use independent duplicate screening, which may have introduced a risk of selection bias. Fifth, our search strategy focused primarily on major biomedical databases. We did not search regional Asian databases or gray literature, such as conference proceedings or trial registries. This may have limited the comprehensiveness of our synthesis. We also excluded studies that didn’t have an extractable effect estimate for quantitative pooling. Finally, we did not register this review in PROSPERO in advance. We also did not perform a formal certainty-of-evidence assessment using GRADE because of the limited number of studies and considerable heterogeneity. However, we strongly encourage prospective registration and GRADE assessment in future large-scale reviews.

Despite these limitations, the included studies were generally of high quality, and sensitivity analyses confirmed that the results were consistent across diverse clinical settings. Nonetheless, caution is warranted when interpreting these findings.

## Conclusions

Overall, our findings suggest that a higher baseline NLR may be associated with increased mortality risk among IPF patients, although the association did not reach statistical significance within the primary adjusted analysis. A supplementary analysis, which included an additional study with an unadjusted HR, suggested a similar direction of association. However, adjusted and unadjusted estimates were combined, so these findings are exploratory and should be interpreted with caution. Given its affordability and accessibility, NLR warrants further investigation in larger prospective multicenter studies to more accurately determine its role in prognostic evaluation and therapeutic decision-making.

## Figures and Tables

**Figure 5:**
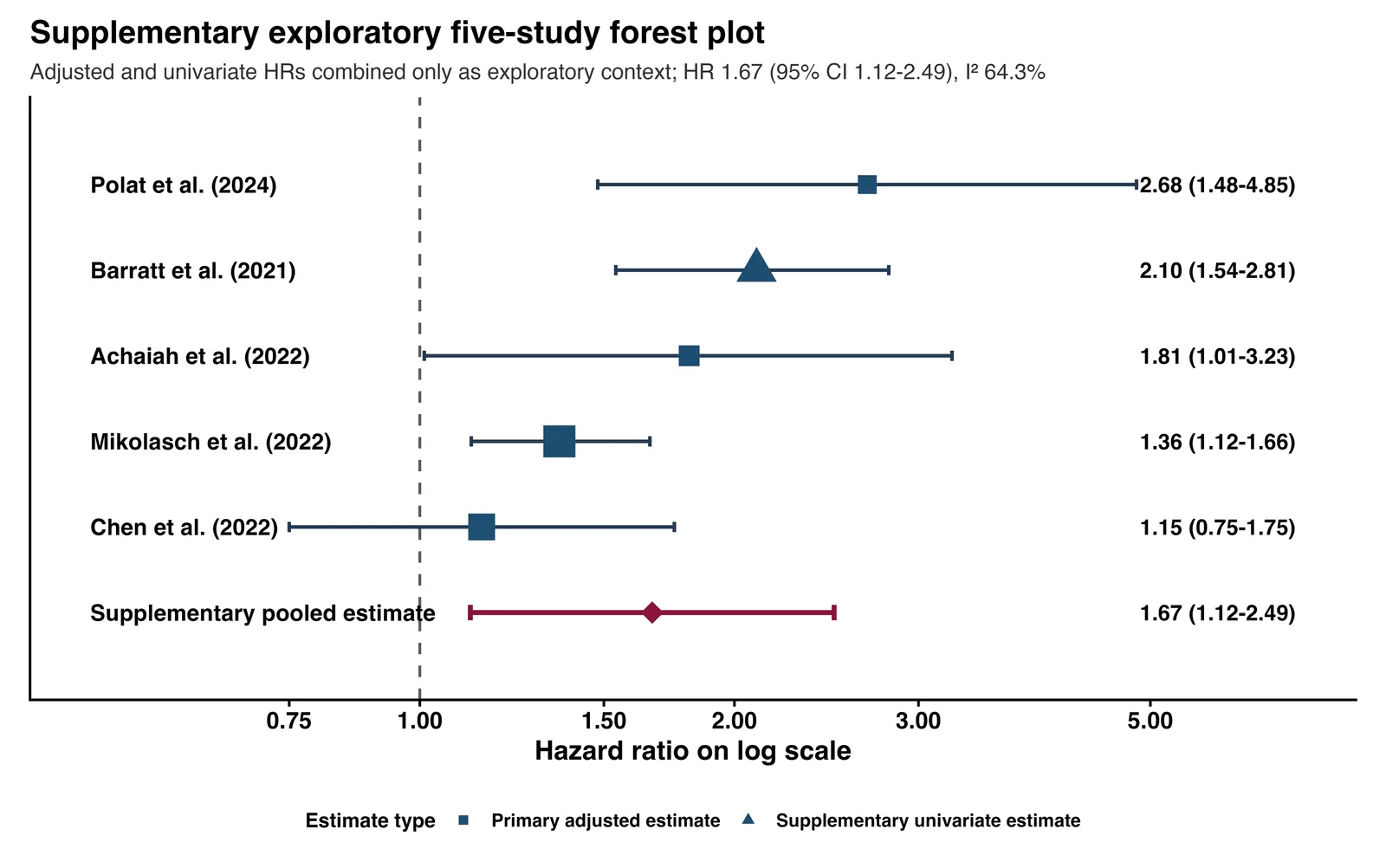
Forest plot of the supplementary exploratory five-study meta-analysis. This analysis combined four adjusted hazard ratios from Mikolasch et al. [18], Polat et al. [19], Achaiah et al. [20], and Chen et al. [21], along with one univariate hazard ratio from Barratt et al. [22].

**Figure 6:**
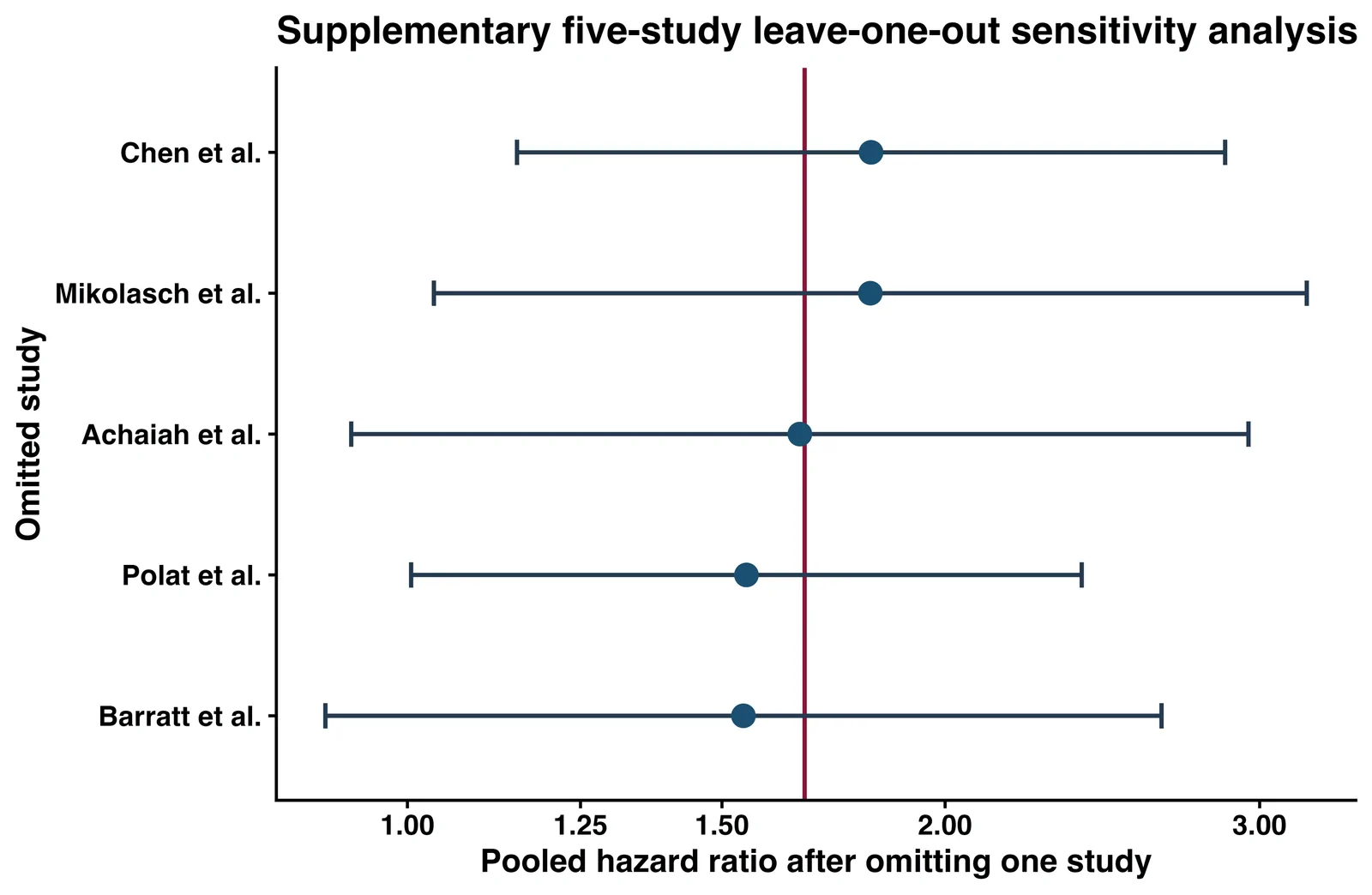
Leave-one-out sensitivity analysis of the supplementary five studies. The analysis included Mikolasch et al. [18], Polat et al. [19], Achaiah et al. [20], Chen et al. [21], and Barratt et al. [22].

**Figure 7:**
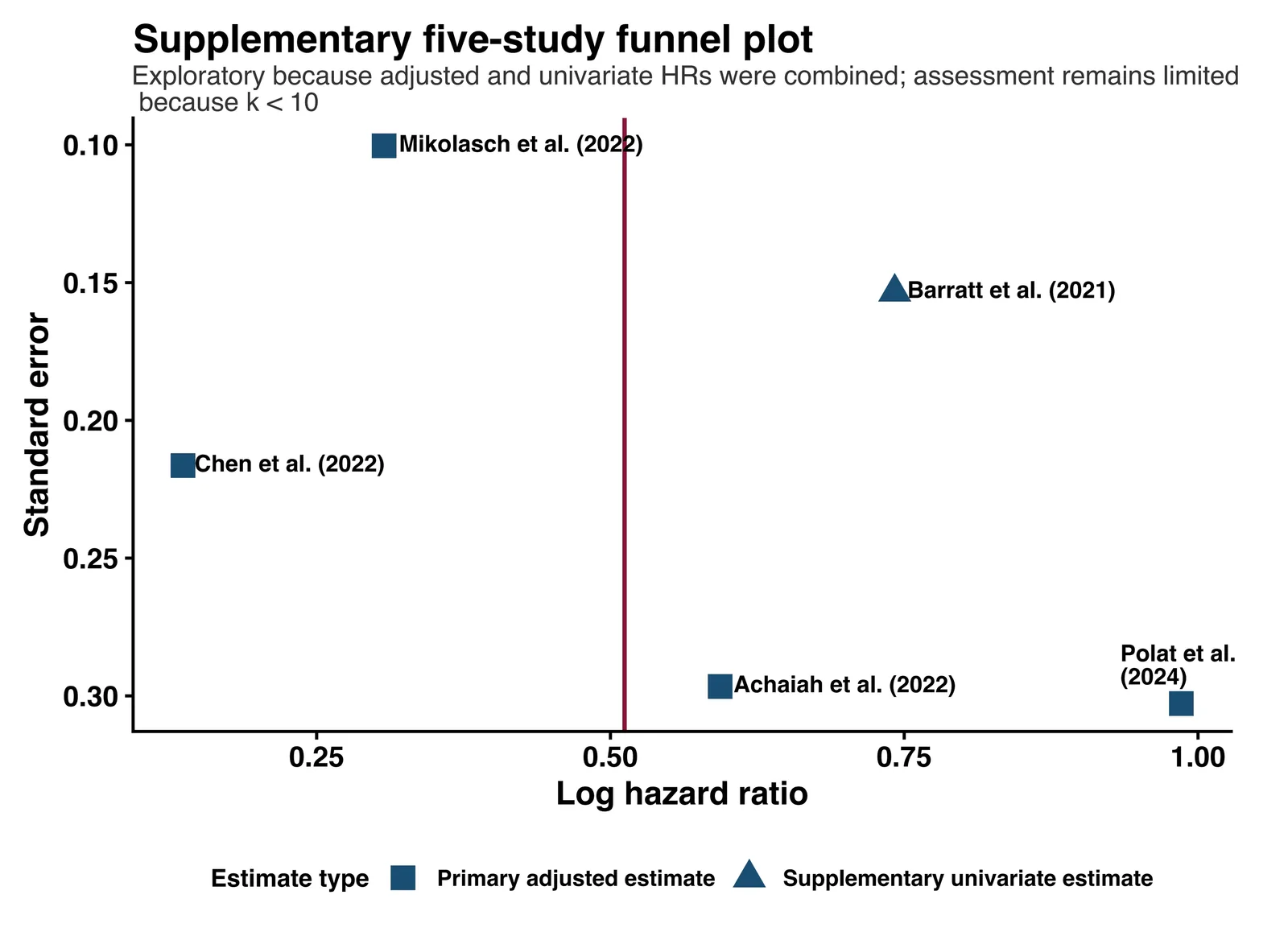
Funnel plot of the supplementary five-study meta-analysis. Studies included: Mikolasch et al. [18], Polat et al. [19], Achaiah et al. [20], Chen et al. [21], and Barratt et al. [22]. Interpretation is limited because the analysis is exploratory and fewer than 10 studies were included.

## Appendices

Appendix A 

Appendix B  

Appendix C  

**Table 7:** Results of the leave-one-out sensitivity analysis for the supplementary five studies.

Omitted study	Pooled HR (95% CI)	p-value	I² (%)
Mikolasch et al. [18]	1.82 (1.03−3.19)	0.043	57.7
Polat et al. [19]	1.55 (1.00−2.39)	0.049	61.3
Achaiah et al. [20]	1.66 (0.93−2.96)	0.069	74.6
Chen et al. [21]	1.82 (1.15−2.87)	0.025	62.8
Barratt et al. [22]	1.54 (0.90−2.64)	0.083	55.1

Appendix D  
